# Biomechanical finite element methods study of a novel internal fixation system for lumbar spondylolysis

**DOI:** 10.3389/fbioe.2026.1718639

**Published:** 2026-06-05

**Authors:** Jingyuan Li, ChengFei Du, Yulei Wang, Di Du, Jinlong Liang, Nengqi Shao, Fanzhe Feng, Wenhao Xu, Yongqing Xu, Yi Cui

**Affiliations:** 1 Department of Orthopedics and Trauma, The First Affliated Hospital of Xinjiang Medical University, Urumqi, Xinjiang, China; 2 Tianjin University of Technology, Tianjin, China; 3 Second People’s Hospital of Qujing City, Qujing, China; 4 920th Hospital of People’s Liberation Army Joint Logistic Support Force, Kunming, China; 5 Dali University, Dali, China; 6 Department of Orthopaedic Surgery, 920th Hospital of Joint Logistics Support Force, Kunming, Yunnan, China

**Keywords:** biomechanical, finite element analysis, internal fixation system, lumbar spondylolysis, surgical therapy

## Abstract

**Objectives:**

This study aimed to evaluate the biomechanical efficacy of a novel internal fixation system for the treatment of lumbar spondylolysis (LS). In addition, the changes in the mechanical performance of the proposed fixation construct during progressive compression were systematically investigated.

**Methods:**

A healthy 25-year-old male volunteer was recruited for lumbar spine CT data acquisition to construct and validate a nonlinear finite element model of the L4-S1 spinal segment (A); Based on this, models were established for L5 spondylolysis (B); L5 spondylolysis model with traditional internal fixation (C); L5 spondylolysis model with the pressurization process of the novel LS repair device fixation (D→E→F). For these six models, we constrained the lower surface of the S1 vertebral body while applying an axial compression force of 500 N and a moment load of 7.5 N m on the upper surface of the L4 vertebral body to simulate six motions of the lumbar spine. The performance of each finite element model was evaluated by comparing the range of motion (ROM), maximum displacement, and maximum pressure experienced by the lumbar spine under different motion conditions.

**Results:**

Compared with Model C, Models D, E, and F exhibited a reduced ROM and maximum displacement under left axial rotation and right axial rotation conditions. Notably, compared with Model C, the novel internal fixation models consistently demonstrated a decreasing trend in the maximum stress on the intervertebral discs (IVD) and an increasing trend in the maximum stress on the articular cartilage and maximum stress and displacement of the bone graft. Moreover, The progressive pressurization (D→E→F) of the novel internal fixation model further enhanced stress transfer between the isthmic defect ends and the bone graft.

**Conclusion:**

Compared with the conventional fixation model (Model C), the novel internal fixation models (Models D, E, F) provided superior spinal stability, more effectively restored physiological stress levels in the facet cartilage, and generated greater mechanical stimulation within the bone graft region. These findings suggest that the proposed fixation system may provide a more favorable biomechanical environment for pars defect healing and graft fusion.

## Introduction

Lumbar spondylolysis (LS) is a common orthopedic condition that predominantly affects adolescents and young adults (Debnath, 2021; Ambati et al., 2015). It is characterized by a defect of the pars interarticularis, the bony region located between the superior and inferior articular processes of the lumbar vertebral arch (Leone et al., 2011). The reported incidence of LS is approximately 6%, with a higher prevalence among individuals engaged in high-intensity sports and military training (Sakai et al., 2010; Schwartz et al., 2018; Selhorst et al., 2019). The pars interarticularis plays a critical role in maintaining the balance between anterior and posterior shear forces acting on the vertebral body. Disruption of this structure reduces resistance to anterior shear loading and compromises the mechanical stability of the lumbar spine (Xing et al., 2019; Campbell et al., 2016). Consequently, LS may impair lumbar spine function and impose abnormal mechanical stress on adjacent vertebral bodies and intervertebral discs (IVDs), thereby accelerating degenerative changes (Patel et al., 2024). In more severe cases, LS may lead to irreversible complications, including nerve injury and lumbar intervertebral disc degeneration, significantly affecting patients’ daily activities and quality of life. Due to anatomical and biomechanical factors, LS most frequently occurs at the L5 level, followed by the L4 level, with the L4–L5 segment representing one of the most commonly affected regions (Hsu and Jenkins, 2017; Mohile et al., 2022).

At present, symptomatic LS is primarily managed with conservative treatment, particularly in patients with low-grade spondylolisthesis. For patients with persistent symptoms following 6–12 months of unsuccessful conservative management, the diagnosis shifts to chronic isthmic defect, and prompt surgical treatment is indicated (Choi et al., 2022; Malas et al., 2022; Mohile et al., 2022). Current surgical strategies for LS mainly include single-segment and multisegment fixation, both of which have demonstrated favorable clinical outcomes (Jain and Khan, 2019; Zhang et al., 2021; Choi et al., 2022). Although intersegmental fusion can also be applied, it is less commonly used in patients without severe spondylolisthesis due to its restriction of spinal mobility and the potential risk of adjacent segment degeneration (Gao et al., 2023; Dhar et al., 2025). Therefore, the single-segment fixation technique is recommended for patients under 30 years of age with no significant spondylolisthesis or intervertebral disc degeneration. Among these techniques, the pedicle screw–vertebral plate hook (PSVPH) and pedicle screw-U-shaped rod (PSUSR) systems are widely used for direct repair of the pars defect. The PSUSR system has minimal impact on spinal motion and is considered technically straightforward, with satisfactory clinical outcomes in pain relief (Li et al., 2022; Haidong, 2021). However, a potential limitation of this technique is the increased risk of spinous process fracture due to excessive compressive loading (Yurac et al., 2021; Ye et al., 2022).

Moreover, the pedicle screw–vertebral plate hook (PSVPH) fixation technique, which promotes bone healing by applying compressive force across the pars defect using compression forceps, is among the most commonly employed methods. However, the magnitude of compression largely depends on the surgeon’s experience and remains difficult to standardize or precisely control. An optimal level of compression facilitates defect closure and promotes bone healing. In contrast, insufficient compression may fail to achieve adequate apposition of the defect ends, resulting in delayed union or even nonunion in some cases (Li et al., 2022; Ye et al., 2022). Conversely, excessive compression may increase stress transmission to adjacent structures, potentially accelerating intervertebral disc degeneration (Yurac et al., 2021; Geiger and Wirries, 2019).

**FIGURE 1:**
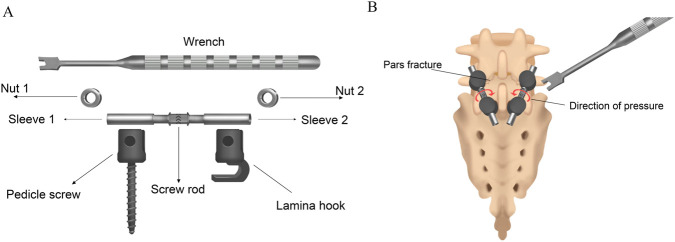
Diagrams of the novel internal fixation system. **(A)** Components of the novel internal fixation system. **(B)** Bilateral pars fractures at the L5 level treated with a novel internal fixation system.

To achieve controllable compression across the pars interarticularis defect and thereby promote bone healing, we designed and developed a novel internal fixation system, as illustrated in Figure 1. This system consists of pedicle screws, laminar hooks, sleeves, threaded rods, and locking nuts, with an additional wrench mechanism that enables precise regulation of the applied compressive force during defect repair (Figure 1A). Furthermore, the modified rod is compatible with minimally invasive surgical approaches. In preliminary *in vitro* experiments, this novel construct demonstrated greater axial gripping strength compared with conventional spinal rods and was capable of meeting the mechanical demands of the human pars interarticularis. These findings suggest that the proposed system may serve as a promising alternative to traditional fixation techniques for the treatment of LS (Li et al., 2024; Jain and Khan, 2019). However, despite these encouraging results, the biomechanical effects of this device on lumbar spondylolysis repair, particularly its ability to achieve effective and stable compression across the defect site, remain unclear.

Therefore, the present study aimed to quantitatively evaluate the biomechanical performance of the proposed fixation system using finite element analysis, to compare it with conventional fixation techniques, and to investigate the effects of progressive compression on spinal biomechanics. To date, the surgical implantation procedure of this novel system has not been clearly described in the existing literature.

## Materials and methods

### Reconstruction of lumbosacral models

An intact finite element (FE) model of the L4-S1 spinal segments (Model A) was constructed based on a previously developed and validated lumbar spine model (L1-S1), as reported by Du et al. (2016), Du et al. (2021), Ren et al. (2023). Detailed procedures for model construction, validation, and convergence analysis have been described in these studies. The intact L4–S1 FE model included cancellous bone, cortical bone, endplates, posterior elements, intervertebral discs, and surrounding ligaments. The intervertebral disc was modeled as consisting of a nucleus pulposus and an annulus fibrosus, accounting for approximately 44% and 56% of the total disc volume, respectively. Collagen fibers embedded within the annulus fibrosus were oriented at an average angle of ±30° relative to the endplates (Sun et al., 2025; Shirazi-Adl et al., 1986).

Initially, 2 mm of external threading was retained at both ends of the construct. The novel spinal rod has a diameter of 6 mm and incorporates a central screw with bidirectional (counterclockwise) threads on both sides. The central screw has an outer diameter of 4.5 mm, an inner diameter of 3.2 mm, and a length of 8 mm. In addition, a rotating mechanism is located at the center of the screw, with a length of 8 mm. Sleeves are positioned on both sides of the screw, with one side designed as a non-threaded solid segment to ensure structural stability (Figure 1A). The threads at the interface between the sleeve and the screw rod were removed to facilitate direct contact between these components. The relative motion between the sleeve and the screw rod is regulated by a cylindrical connector, which permits both axial displacement and rotational movement. During operation, compressive force is generated by rotating the central screw using a wrench, thereby driving the sleeves on both sides toward the screw. This mechanism enables controlled axial compression across the pars interarticularis. Both the screws and the novel spinal rod are manufactured from titanium alloy (Figure 1B). During the procedure, the lamina and facet joints were adequately exposed. Fibrous tissue at the pars interarticularis defect was debrided, followed by bone grafting. Pedicle screws were then inserted, and laminar hooks were subsequently placed. The bilateral sleeves were connected to the screw rods and assembled onto the laminar hooks and pedicle screws. After tightening the locking nuts, compression across the defect site was achieved by rotating the screw rods using a wrench. In contrast to conventional spinal rod systems, the proposed device enables precise and controllable compression at the fracture ends of the pars interarticularis.

A healthy 25-year-old male volunteer was recruited for imaging data acquisition. Computed tomography (CT) scans were performed using a 64-slice spiral CT scanner (GE Healthcare, USA). The scanning parameters were set as follows: tube voltage of 140 kV, tube current of 525 mA, and slice thickness of 0.625 mm. The image resolution was 512 × 512 pixels. This study was reviewed and approved by the Ethics Committee of the 920th Hospital of the Joint Logistic Support Force of the Chinese People’s Liberation Army.

**FIGURE 2:**
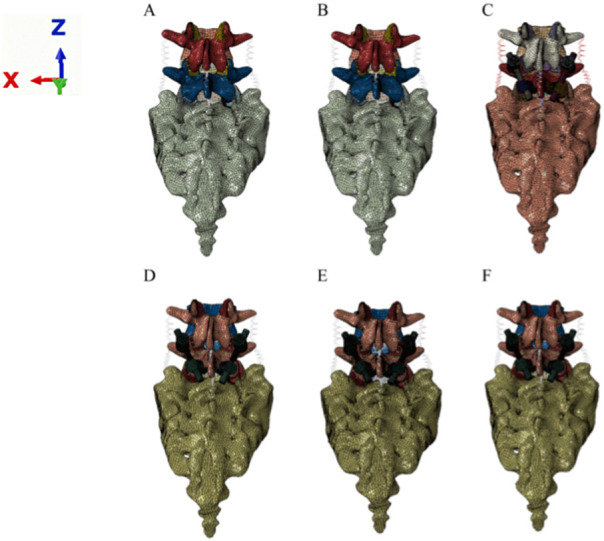
Six finite element models **(A)** intact model **(B)** spondylolysis model **(C)** traditional internal fixation model **(D)** the novel internal fixation with a unilateral sleeve advancement towards the screw by 0 mm **(E)** the novel fixation with a unilateral sleeve advancement towards the screw by 1 mm **(F)** the novel fixation with a unilateral sleeve advancement towards the screw by 2 mm.

Six FE models were established in this study. A global Cartesian coordinate system was defined for the spinal model, in which the X-axis represented the mediolateral direction, the Y-axis the anteroposterior direction, and the Z-axis the cephalocaudal direction (Figure 2). Model A: Original model of CT scan showed intact L4-S1 vertebral model with normal L5 vertebral body; Model B: bilateral spondylolysis of L5 without internal fixation; Model C: Traditional internal fixation on the L5 pars interarticulars of Model B; Model D: The novel device fixation featured a unilateral sleeve advancement toward the screw by 0 mm on the L5 pars interarticulars of Model B; Model E: The novel device fixation featured a unilateral sleeve advancement toward the screw by 1 mm on the L5 pars interarticulars of Model B; Model F: The novel device fixation featured a unilateral sleeve advancement toward the screw by 2 mm on the L5 pars interarticulars of Model B.

### Material properties

**FIGURE 3:**
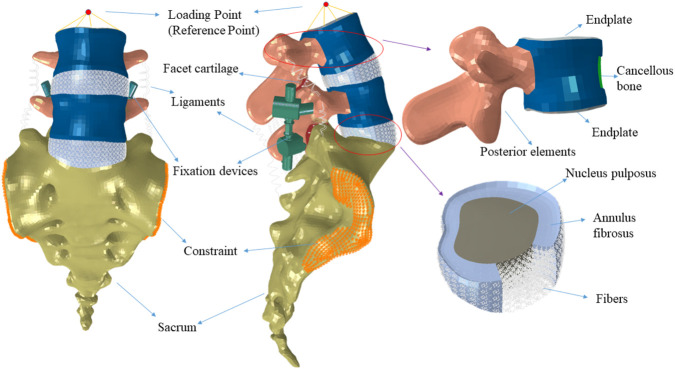
Finite element model of the lumbar spine showing structural components and boundary conditions.

A detailed illustration of the lumbar finite element model, including its structural components, is provided in Figure 3. The model consists of vertebral bodies, intervertebral discs (including nucleus pulposus, annulus fibrosus, and fibers), posterior elements, ligaments, facet cartilage, and fixation devices, as listed in Table 1 (Ambati et al., 2015; Wang et al., 2021; Li et al., 2022; Gao et al., 2023; Dhar et al., 2025).

**TABLE 1:** Material properties and element types of the finite element models (Ambati et al., 2015; Wang et al., 2021; Li et al., 2022; Gao et al., 2023).

Component	Constitutive model	Material parameters	Element type
Cortical bone	Linear elastic	E = 12,000 MPa, ν = 0.3	Hexahedral
Cancellous bone	Linear elastic	E = 100 MPa, ν = 0.3	Tetrahedral
Endplate	Linear elastic	E = 10,000 MPa, ν = 0.4	Hexahedral
Posterior bony elements	Linear elastic	E = 3,500 MPa, ν = 0.25	Tetrahedral
Sacrum	Linear elastic	E = 5,000 MPa, ν = 0.2	Tetrahedral
Facet cartilage	Neo-hookean	C10 = 2 MPa	Hexahedral
Annulus fibrosus	Mooney–Rivlin	C10 = 0.18 MPa, C01 = 0.045 MPa	Hexahedral
Nucleus pulposus	Mooney–Rivlin	C10 = 0.12 MPa, C01 = 0.03 MPa	Hexahedral
Annulus fibers	Nonlinear	Stress–strain relationship	spring
Ligaments	Nonlinear	Force–displacement relationship	spring
Instrumentation	Linear elastic	E = 110,000 MPa, ν = 0.3	Tetrahedral

The vertebrae and intervertebral discs were discretized using brick elements. The annulus ground substance and nucleus pulposus were modeled as nearly incompressible, hyperelastic materials (Shirazi-Adl et al., 1986; Du et al., 2014; Gouveia et al., 2025). The spinal ligaments and annulus fibrosus fibers were represented by tension-only spring elements with nonlinear mechanical properties derived from the literature (Shirazi-Adl et al., 1986; Tao et al., 2024). Additionally, linear contact relationships were set for the facet joints and the interaction between the hook and vertebrae, and the bonding mode was set to the interface of the screw and plate, screw, and bone.

### Loading and boundary conditions

A reference node was defined near the center of the superior surface of L4 and was kinematically coupled to the entire superior endplate of L4 (Figure 3). To simulate physiological loading conditions of the human spine, a follower load of 500 N was applied along the curvature of the spinal column. In addition, a pure moment of 7.5 Nm was applied to the reference node in different directions to replicate six physiological motions: flexion, extension, left lateral bending (LLB), right lateral bending (RLB), left axial rotation (LAR), and right axial rotation (RAR) (Du et al., 2015; Cucinotta et al., 2024). During loading, the surfaces of sacro-iliac joints were constrained in all directions.

### Primary observation indicators

Under six conditions, namely, flexion, extension, left bending, right bending, left axial rotation, and right axial rotation, the range of motion (ROM), maximum displacement, and maximum stress distribution characteristics were evaluated and compared among the six groups of models. The ROM was calculated as the angular rotation around the *X*, *Y*, and *Z*-axes, representing flexion-extension, lateral bending, and axial rotation, respectively.

## Results

### Range of motion

**FIGURE 4:**
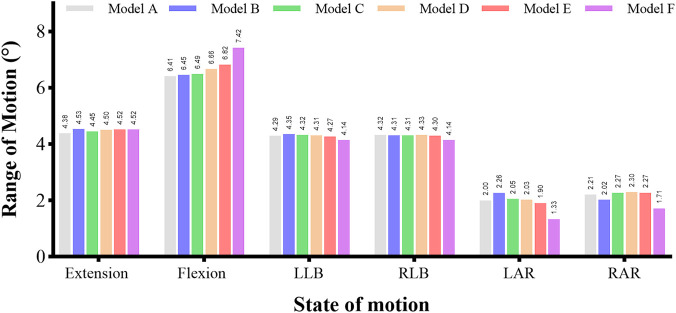
The ROM of Model A, B, C, D, E and F in six postures of motion.

The ROM results for the six models are presented in Figure 4. Compared with the intact Model A, the Model B exhibited increased ROM in extension, flexion, LLB, and LAR postures, with a maximum increase of 0.26° in LAR and a minimum increase of 0.04° in flexion. Following the implantation of traditional internal fixation Model C, there was a decrease in ROM in all postures except for flexion, RLB and RAR. Compared with Model C, the novel fixation models (Models D, E, and F) demonstrated further reductions in ROM under LLB and LAR conditions, while an increasing trend was observed in flexion and extension. Specifically, Model D showed a slight reduction (approximately 1%) in LAR only, whereas Model E exhibited reductions ranging from 0.7% to 7.3%, and Model F demonstrated more pronounced decreases ranging from 3.7% to 35.1%. Overall, except for flexion and extension, a progressive decrease in ROM was observed from Models D to F, indicating enhanced segmental stability with increasing compression. Among these, Model F exhibited the lowest ROM values, suggesting the greatest stabilizing effect (Choi et al., 2022).

### Model displacement analysis

**FIGURE 5:**
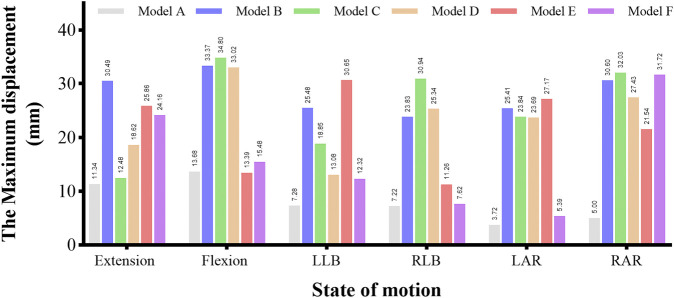
The maximum displacement of Model A, B, C, D, E and F in six postures of motion.

The displacement distribution of the models was analyzed to further evaluate the effects of internal fixation on vertebral stability. Compared with the intact Model A, Model B exhibited a marked increase in maximum displacement, ranging from 144% to 583% across all six motion conditions. The greatest increase was observed during LAR, whereas the smallest increase occurred during flexion (Figure 5). Compared with Model B, the maximum displacement of Models C, D, E, and F decreased under extension. However, under certain motion conditions, the maximum displacement in these fixation models remained higher than that of the Model A (Figure 5). Relative to Model B, Model C demonstrated notable reductions in maximum displacement, particularly under extension and LLB conditions. Furthermore, the Models D, E, and F exhibited more pronounced reductions in maximum displacement across most motion conditions. However, limited changes were observed in RLB for Model D, right axial rotation (RAR) for Model E, and LAR for Model F. Overall, the novel fixation system demonstrated a more effective displacement-limiting capability, with a trend toward enhanced stability under progressive compression.

### Maximum stress of the IVD

**FIGURE 6:**
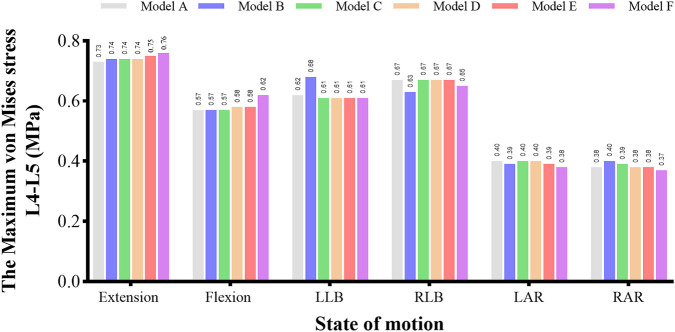
The maximum von Mises stress of L4-L5 IVD of Model A, B, C, D, E and F in six postures of motion.

**FIGURE 7:**
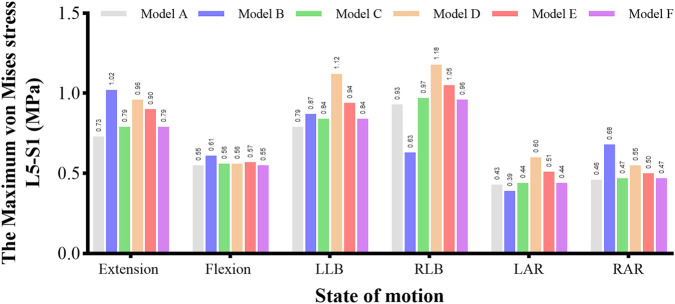
The maximum von Mises stress of L5-S1 IVD of Model A, B, C, D, E and F in six postures of motion.

Among Models A-F, no significant differences were observed in the maximum stress of the L4-L5 intervertebral disc under most motion conditions, except for Model B during LLB (Figure 6). As shown in Figure 7, no substantial differences in maximum stress were detected among the six models during flexion of the L5-S1 intervertebral disc. However, under RLB and LAR, the maximum stress values in the L5-S1 disc for both the Model C and the Models D, E and F were higher than those observed in Model B. Specifically, Model D exhibited increases of 28.7%, 87.3%, and 53.8% under LLB, RLB, and LAR, respectively. In contrast, during extension and RAR, all fixation models C, E, and F demonstrated reduced maximum stress compared with Model B, with average decreases of 15.7% and 26.8%, respectively. Furthermore, compared with the Model C, novel internal fixation Models D, E and F exhibited a gradual reduction in stress magnitude under these conditions, indicating a trend toward improved stress distribution with progressive compression.

### Maximum stress of articular cartilage

**FIGURE 8:**
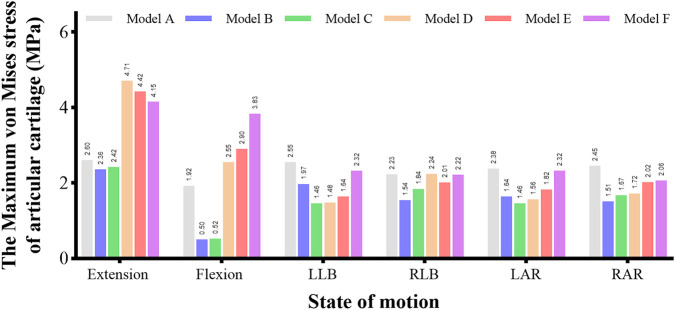
The maximum von Mises stress of articular cartilage of Model A, B, C, D, E and F in six postures of motion.

The average maximum stresses of the articular cartilage in Models A-F under six different motion postures were 2.36 ± 0.25 MPa, 1.59 ± 0.62 MPa, 1.56 ± 0.62 MPa, 2.38 ± 1.22 MPa, 2.47 ± 1.05 MPa and 2.82 ± 0.92 MPa, respectively (Figure 8). Compared with the Model A, Model B exhibited a reduction in articular cartilage stress, resulting in values comparable to those of Model C. However, Models D, E and F demonstrated increased articular cartilage stress relative to Model B, with increments of 49.8%, 55.6%, and 77.5%, respectively (D < E < F). This trend may be attributed to the impaired load-bearing capacity of the articular cartilage following spondylolysis. Compared with the conventional fixation system (Model C), the novel fixation system appears to restore load transmission to the posterior elements, thereby partially recovering the physiological stress distribution of the articular cartilage.

### Maximum stress and displacement of the bone graft

**FIGURE 9:**
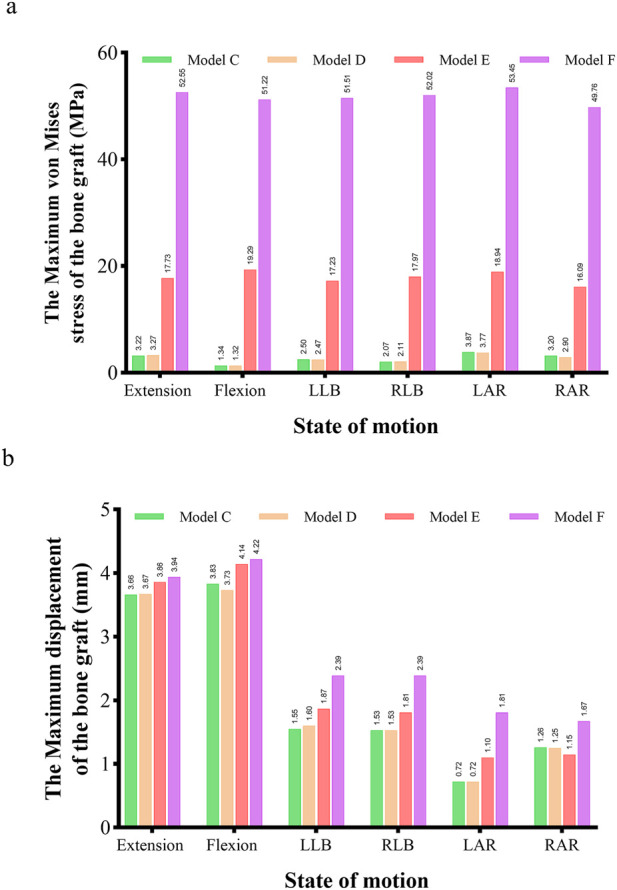
The maximum von Mises Stress and maximum displacement of bone graft of Model C, D, E and F in six postures of motion. **(a)** The maximum von Mises Stress of Model C, D, E and F in six postures of motion; **(b)** The maximum displacement of bone graft of Model C, D, E and F in six postures of motion.

The biomechanical environment of the bone graft is a critical factor in evaluating the efficacy of internal fixation systems for spondylolysis repair. As shown in Figure 9a, compared with Model C, the novel fixation models E and F exhibited substantially higher maximum stress within the bone graft region, whereas Model D showed no evident increase. Specifically, under all six motion conditions, Models E and F demonstrated stress increases ranging from 403% - 1,340% and 1,281%–3,722%, respectively (E < F). In addition, both Models E and F exhibited greater displacement of the bone graft compared with Model C across all motion conditions, with increases ranging from 5% - 53% and 8%–151%, respectively (Figure 9b). These findings indicate that progressive compression enhances stress transfer to the bone graft region, which may promote graft-bone interface stimulation and potentially facilitate bone healing. This compression-dependent increase in graft stress suggests a potential mechanical advantage of the novel fixation system in promoting pars defect healing.

### Maximum stress and stress distribution of the isthmus

**FIGURE 10:**
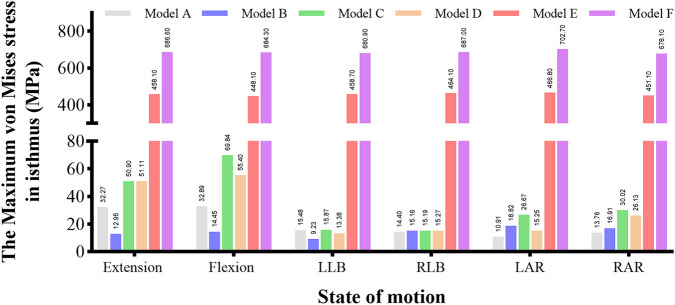
The maximum von Mises stress on isthmus of Model A, B, C, D, E and F in flexion, extension, LLB, RLB, LAR and RAR.

**FIGURE 11:**
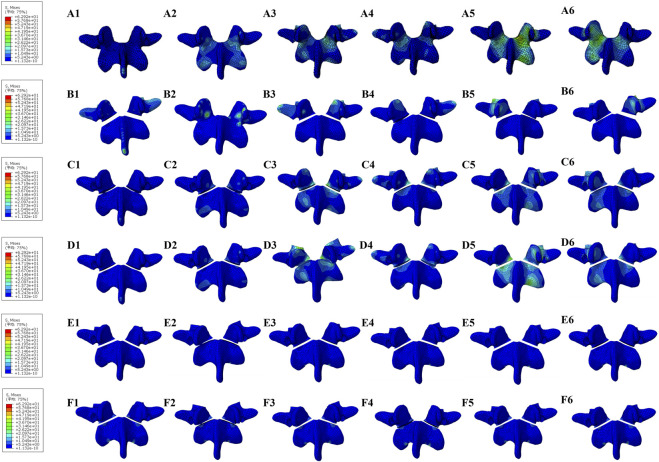
Stress nephograms of isthmus in Model A, B, C, D, E and F in flexion, extension, LLB, RLB, LAR and RAR (A1-A6) The stress distribution of isthmus in Model A in six postures of motions (B1-B6) The stress distribution of isthmus in Model B in six postures of motions (C1-C6) The stress distribution of isthmus in Model C in six postures of motions (D1-D6) The stress distribution of isthmus in Model D in six postures of motions (E1-E6) The stress distribution of isthmus in Model E in six postures of motions (F1-F6) The stress distribution of isthmus in Model F in six postures of motions.

In the intact model (Model A), as shown in Figure 10, the maximum stress at the pars interarticularis decreased during lateral bending and axial rotation compared with flexion and extension. In contrast, in Model B, stress at the defect site decreased under flexion and extension but increased under lateral bending and axial rotation relative to Model A. Moreover, the stress distribution shifted from a relatively uniform pattern in the pars interarticularis region (Model A) to a more localized distribution at the bilateral defect sites (Model B). In Model C, the maximum stress increased across all motion conditions, particularly during extension and flexion, reaching values of 50.9 MPa and 69.84 MPa, respectively (Figure 10). Stress distribution analysis showed a relatively uniform pattern during flexion and extension, whereas stress concentration persisted at the bilateral defect regions during lateral bending and axial rotation (Figure 11). Compared with Model C, the Models D, E, F) demonstrated distinct stress modulation patterns. Model D showed maximum stress values comparable to those of Model C, whereas Models E and F exhibited substantial increases, with stress elevations ranging from 542% to 2,955% for Model E and from 880% to 4,423% for Model F (E < F) (Figure 10). Furthermore, stress distribution in Model D remained relatively dispersed across the bilateral pars defects, while Models E and F demonstrated a broader and more homogeneous stress distribution over the articular surfaces without evident stress concentration (Figure 11). These findings suggest that progressive compression promotes stress redistribution at the pars interarticularis, potentially creating a more favorable mechanical environment for defect stabilization and healing.

### Stress analysis of the internal fixation system

**FIGURE 12:**
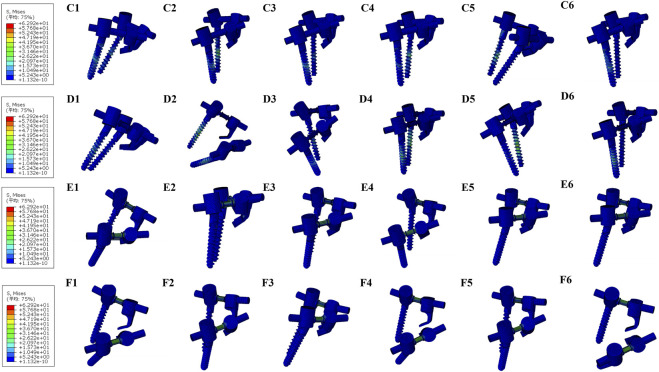
Stress nephogram for model C, D, E, F in flexion, extension, LLB, RLB, LAR and RAR (C1-C6) Model C (D1-D6) Model D (E1-E6) Model E (F1-F6) Model F.

**FIGURE 13:**
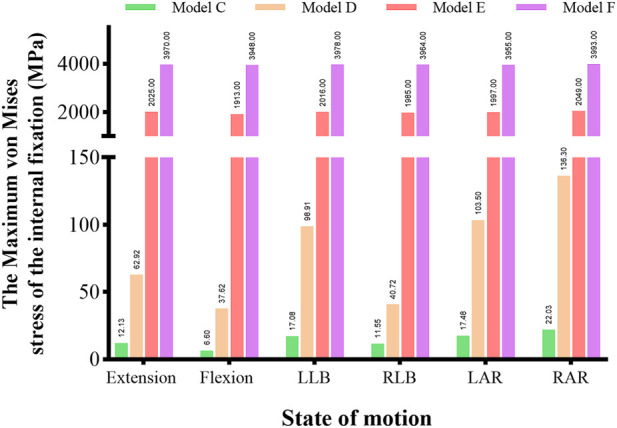
The maximum von Mises Stress in the internal fixation system of model C, D, E, F in flexion, extension, LLB, RLB, LAR and RAR.

The mechanical strength of the internal fixation system is a critical parameter in spinal surgery. The stress nephograms of the Model C and the Models D, E and F are presented in Figure 12, while the corresponding maximum von Mises stresses under six motion conditions are summarized in Figure 13. In Model C, stress was primarily concentrated on the left pedicle screw, with the maximum value occurring during RAR, reaching 22.03 MPa (Figures 12, 13). In contrast, Models D, E and F exhibited peak stresses during RAR, with maximum values of 136.3 MPa, 2049 MPa, and 3,993 MPa, respectively. In Model D, stress was mainly localized within the screw itself, whereas in Models E and F, stress concentration shifted to the junction between the screw and the spinal rod, accompanied by a more distributed stress pattern throughout the construct. Across all motion conditions, the maximum stresses observed in Models D, E and F were consistently higher than those in the conventional fixation model (Model C). Notably, Model F demonstrated markedly elevated peak stress values, approaching 4,000 MPa under multiple loading conditions.

These findings indicate that increasing compression is associated with a substantial rise in implant stress, particularly at critical junctional regions. The potential biomechanical implications of these elevated stress levels are further discussed in the Discussion section.

## Discussion

Previous studies have extensively investigated the biomechanical performance of various internal fixation techniques for pars repair using computer simulation and finite element analysis. For instance, Sairyo et al. evaluated the effects of Buck direct repair on intervertebral disc stress using a finite element model (Sairyo et al., 2006; Virk et al., 2022). Li et al. conducted a three-dimensional finite element comparison between the pedicle screw-U-rod system and the pedicle screw-lamina hook system for the treatment of lumbar spondylolysis (Li et al., 2022; Shirazi-Adl et al., 1986). In addition, Ye et al. analyzed the biomechanical differences between Buck screw fixation alone and Buck screw fixation combined with intersegmental fixation (Ye et al., 2022; Pei et al., 2023). Despite these advances, most existing studies have primarily focused on comparisons among different fixation constructs, with limited attention given to the role of controlled compression across the pars defect. In the present study, we developed a novel modified pedicle screw-hook-rod fixation system based on a conventional construct, with the aim of achieving precise and controllable compression at the defect site. This design was intended to address the limitation of insufficient compression associated with existing internal fixation techniques.

In this study, six three-dimensional finite element models of L4-S1 were constructed to explore the biomechanical effect of a novel internal fixation system for LS repair. Compared with the other models, the spondylolysis model (Model B) exhibited a slight increase in ROM, particularly under LAR, indicating reduced segmental stability. This finding is consistent with the results of Yabuno et al. and confirms that internal fixation can effectively reduce the spinal ROM and minimize movement-related trauma (Yabuno et al., 2019). Following placement of the traditional internal fixation model (Model C), the vertebral ROM was well restored in four motion postures, but not for flexion or the RAR, approaching the normal levels observed in Model A. These results suggested that traditional internal fixation has a certain efficacy in restoring the vertebral ROM. Comparatively, because of the compressive pressure on the fracture end of the isthmus (D→E→F), novel internal fixation Models D, E, and F demonstrated varying degrees of reduction in vertebral ROM during LLB, RLB, and LAR postures compared to Model C. Model C (3.98°) demonstrates a superior average activity in contrast to Model F (3.88°). These results suggest that the novel fixation system provides a compression-dependent stabilization effect, which may be beneficial for reducing micromotion at the defect site while preserving overall spinal function (Wang et al., 2021).

In terms of displacement, the postoperative maximal displacement in the novel fixation models D,E and F remained higher than that of the intact model and was comparable to the lesion model. Although partial reductions were observed under certain loading conditions, full restoration of normal displacement was not achieved. This may be due to the persistent discontinuity of the pars interarticularis and the absence of simulated bone healing in the finite element model. These findings indicate that mechanical fixation alone improves stability but may not fully recover the native biomechanical behavior without biological repair. In addition, the mean displacement of Model C (25.49 mm) surpassesd that of Model F (16.12 mm). This indicates that when the novel internal fixation system was compressed to 2 mm, the lumbar spine exhibited greater stability compared with the traditional internal fixation system, culminating in an enhancement of the early postoperative stability for patients utilizing the new internal fixation system (model F) (Cai et al., 2020).

Furthermore, component-level analysis provided additional insights into both biomechanical mechanisms and potential clinical implications. Analysis of IVD stress indicated that no significant differences were observed in the L4-L5 disc among the models under all motion conditions. At the L5-S1 level, both the Model C and the Models D, E and F exhibited reduced IVD stress during extension and RAR compared with the Model B, which may help mitigate excessive disc loading and potentially slow degenerative progression (Castellvi et al., 2007; Zhou et al., 2019). Particularly noteworthy is that compared with traditional internal fixation Model C, Model F showed no substantial difference in L5–S1 intervertebral disc stress. Consequently, after the application of the novel internal fixation, the patient is required to wear a brace for 3 months, as fractures typically heal within this period. Three months later, the patient should undergo a CT scan of the lumbar spine to verify whether the fracture has healed, and the internal fixation can be removed if applicable (Wu et al., 2024). In this study, Model F demonstrated a more diminished average ROM in contrast to Model C, furnishing supplementary evidence that the greater the pressure exerted by the novel internal fixation device, the superior the stability of the lumbar spine. Concerning displacement, all three novel internal fixation models (D, E, and F) as well as Model C demonstrated reduced maximum displacement values compared with those of LS Model B. Notably, the maximum displacement values were lower in Models E and F than in Model C under simulated early postoperative conditions. The relatively reduced maximum displacement signifies the elevated stability of Models E and F. Within models D, E, F, the maximum displacement of model F is relatively smaller, which serves as a guidance for us in clinical practice that the new internal fixation system ought to be compressed to the state of model F to confer greater stability upon the lumbar spine.

In the analysis of articular cartilage, we observed that Model B exhibited a reduction in stress on the articular cartilage due to the spondylolysis, potentially leading to increased load on the spine and peripheral nerves, thereby contributing to additional diseases (Sterba et al., 2018). Notably, Models D, E, and F demonstrated superior stress recovery outcomes comparable to those achieved by Model C. During bone grafting analysis, in comparison to Model C, as the new internal fixation system exerted pressure on the fractured ends of the isthmus (D→E→F), there was increasing micromotion in the bone graft during mobilization. This phenomenon facilitates enhanced blood circulation at fracture sites, thereby promoting healing (Tomlinson and Silva, 2013; Rather et al., 2019). It was also found that with increased pressure applied on the fracture end. Upon the utilization of the new internal fixation (model F), both the maximum stress and displacement of the bone graft exceeded those of the traditional internal fixation (model C), signifying that the new internal fixation system is competent to impart a higher pressure to the isthmus graft and establish a more auspicious fusion environment for it. Simultaneously, the larger graft displacement is conducive to enhancing the blood circulation within the isthmus, thereby facilitating graft fusion (Li et al., 2022; Patel et al., 2024).

After the placement of internal fixation devices, stress may concentrate in the LS. The findings of this study demonstrate that, across all motion postures, the maximum stress observed in traditional internal fixation Model C exceeded that of LS Model B. Compared with Model C, the novel internal fixation Model D effectively reduced the maximum stress. However, it exhibited an uneven distribution of stress in the LLB, RLB, LAR and RAR. When the new internal fixation system underwent the ultimate compression (model F), the stress across the isthmic defect site was substantially higher than that observed with the other fixation constructs, suggesting improved stabilization at the defect ends (Sairyo et al., 2006; Li et al., 2022; Ye et al., 2022). This enhanced compressive stability may facilitate defect healing. The stress within the posterior elements in Models E and F exceeded that observed in Model C, yet the stress was predominantly concentrated at the inferior margin of the lamina, which might be attributed to the compression exerted on the inferior margin of the lamina. In model C, the stress within the lamina was mainly centered at the pars defect during bilateral flexion, extension, and rotation. Nevertheless, in model F, the stress at the pedicle fracture end was more uniformly distributed in all six active directions, which might suggest that the shearing force at the fracture end is lower and is more favorable for the formation of early fibrous tissue healing.

The risk of postoperative loosening and fracture failure of internal fixation devices is dependent on the size and distribution of their maximum stress (Ye et al., 2022; Pan et al., 2023). With respect to implant stress, Model F exhibited markedly elevated localized peak stresses approaching 4,000 MPa under all six loading conditions. These extreme values are most likely attributable to localized stress concentration rather than representing the global mechanical behavior of the fixation system. In finite element analysis, such high peak stresses commonly occur at regions of geometric discontinuity, sharp edges, or contact interfaces, where load transfer is highly localized, particularly in the presence of local mesh refinement. In the present study, the Models D, E and F introduce more complex load transfer pathways and abrupt geometric transitions, which may further amplify local stress accumulation under multidirectional loading conditions. As a result, numerical stress concentrations may arise within a limited number of elements, leading to overestimation of maximum stress values. Similar phenomena have been widely reported in finite element analyses of spinal and orthopedic implants, where localized peak stresses exceed material yield strength without necessarily indicating global structural failure (Adam et al., 2003; Malas et al., 2022). Nevertheless, despite the influence of potential numerical singularities, the consistent observation of elevated stress levels across multiple loading conditions suggests that the Models D, E and F may be more susceptible to stress concentration than conventional designs. This finding indicates a potential mechanical disadvantage in terms of stress distribution. Although *in vitro* experiments have demonstrated that the biomechanical strength of the novel fixation system can meet clinical requirements (Li et al., 2024), they cannot fully capture localized stress accumulation and fatigue-related risks under physiological cyclic loading. Further *in vivo* studies are still warranted to validate its long-term fatigue failure risk and safety. The stress cloud map reveals that in Models C and D, the maximum stress is concentrated on the screw, increasing the risk of screw fracture. Conversely, Models E and F disperse the maximum stress at the screw joint more evenly throughout the screw, thereby reducing the risk of screw fracture (Zhang et al., 2020).

To withstand the localized high stress levels, the selection of materials and the precision of manufacturing processes are paramount. The use of Ti-6Al-4V ELI, characterized by its superior fatigue-to-tensile strength ratio, provides a foundational safety margin (Jawed et al., 2022). Beyond material properties, surface integrity plays a decisive role in fatigue life. Techniques such as shot peening or electropolishing are essential to eliminate surface defects and introduce a compressive stress layer, which acts as a barrier against fatigue crack initiation at high-stress sites (Le Roux et al., 2025).

This study also has certain limitations. First, the utilization of a single lumbar spine in the models may not fully account for interindividual variations, potentially leading to divergent outcomes. Moreover, the examination solely focused on a single case and did not incorporate biological factors, individual differences, and other medical factors that could influence the results of the experimental analysis. Second, the absence of soft tissue models such as muscles in the model partially affects the activity and stress alterations in the lumbar spine. Additionally, the screw threads in the novel spinal rod were ignored, which likely had a minor effect on the computational results. Finally, further verification through the clinical trial is required to ascertain the long-term stability of this novel LS repair device after fixation.

## Conclusion

This study conducted a comparison between the traditional internal fixation system and the novel one under three distinct degrees of compression. We discovered that the novel internal fixation system is more proficient in maintaining the stability of the spine, restoring the stress level of the facet joint cartilage, offering a superior environment for the graft fusion of the pars, and reducing stress concentration at the pars when compressed to the maximum. Nevertheless, the stress and disc stress of the novel internal fixation system are relatively higher than those of the traditional one. However, we have also verified through *in vitro* experiments that its strength is adequate to meet clinical requirements without causing internal fixation fracture. Further *in vivo* studies are required to validate the long-term fatigue resistance and safety of the novel device.

## Data Availability

The original contributions presented in the study are included in the article/supplementary material, further inquiries can be directed to the corresponding author.
